# Segmentation Cues in Conversational Speech: Robust Semantics and Fragile Phonotactics

**DOI:** 10.3389/fpsyg.2012.00375

**Published:** 2012-10-04

**Authors:** Laurence White, Sven L. Mattys, Lukas Wiget

**Affiliations:** ^1^School of Psychology, Plymouth UniversityPlymouth, UK; ^2^Department of Psychology, University of YorkYork, UK; ^3^Department of General Linguistics, University of ZurichZurich, Switzerland

**Keywords:** speech segmentation, semantics, phonotactics, conversational speech, cross-modal priming

## Abstract

Multiple cues influence listeners’ segmentation of connected speech into words, but most previous studies have used stimuli elicited in careful readings rather than natural conversation. Discerning word boundaries in conversational speech may differ from the laboratory setting. In particular, a speaker’s articulatory effort – hyperarticulation vs. hypoarticulation (H&H) – may vary according to communicative demands, suggesting a compensatory relationship whereby acoustic-phonetic cues are attenuated when other information sources strongly guide segmentation. We examined how listeners’ interpretation of segmentation cues is affected by speech style (spontaneous conversation vs. read), using cross-modal identity priming. To elicit spontaneous stimuli, we used a map task in which speakers discussed routes around stylized landmarks. These landmarks were two-word phrases in which the strength of potential segmentation cues – semantic likelihood and cross-boundary diphone phonotactics – was systematically varied. Landmark-carrying utterances were transcribed and later re-recorded as read speech. Independent of speech style, we found an interaction between cue valence (favorable/unfavorable) and cue type (phonotactics/semantics). Thus, there was an effect of semantic plausibility, but no effect of cross-boundary phonotactics, indicating that the importance of phonotactic segmentation may have been overstated in studies where lexical information was artificially suppressed. These patterns were unaffected by whether the stimuli were elicited in a spontaneous or read context, even though the difference in speech styles was evident in a main effect. Durational analyses suggested speaker-driven cue trade-offs congruent with an H&H account, but these modulations did not impact on listener behavior. We conclude that previous research exploiting read speech is reliable in indicating the primacy of lexically based cues in the segmentation of natural conversational speech.

## Introduction

Most studies of speech perception in general, and speech segmentation in particular, have used stimuli elicited in careful readings rather than natural communicative conditions. Conversational speech differs from such laboratory speech along various dimensions that may have consequences for perception. Thus, ensuring the ecological validity of mechanisms established with read stimuli requires corroborative data (for an early example, see Mehta and Cutler, 1988).

Firstly, words in conversational speech tend to be less intelligible than citation forms (e.g., Pickett and Pollack, 1963), with a narrower formant frequency space for vowels, higher rates of vowel reduction and elision, and greater coarticulation and allophonic variation (e.g., Klatt and Stevens, 1973; Brown, 1977; Duez, 1995). Secondly, conversational speech tends to be highly contextualized, with the production and interpretation of utterances potentially dependent on a mutual understanding of the foregoing interaction. In particular, within the phonetic domain, the speaker’s degree of articulatory effort – hyperarticulation vs. hypoarticulation (H&H) – has indeed been held to vary as a function of communicative demands (Lindblom, 1990, 1996). According to this hypothesis, the speaker’s task is to provide minimal but sufficient acoustic information to permit discrimination between those linguistic options permitted by the current context.

With respect to segmentation, listeners exploit multiple sources of information to locate word boundaries in connected speech. Language-specific cues are based on listeners’ experience of the words, rules, and regularities of a particular language, whereas language-general cues arise from potentially universal articulatory adjustments associated with word boundaries – specifically, hyperarticulation/decoarticulation and lengthening (e.g., Tyler and Cutler, 2009). The H&H account of conversational speech production naturally implies that speakers should increase the power of these language-general acoustic-phonetic segmentation cues – through boundary-adjacent hyperarticulation etc. – when information from language-specific cues is lacking. Assuming these acoustic modifications are perceptible to listeners, they should serve to compensate for the local weakness of language-specific cues.

Thus, we examined whether listeners’ exploitation of segmentation cues in conversational speech is subject to a speaker-driven trade-off between language-general and language-specific cues. The results were compared to the same listeners’ behavior when confronted with read speech. We focused on two language-specific sources of word-boundary information, from distinct tiers of Mattys et al. (2005) segmentation hierarchy: semantics (Tier 1) and phonotactics (Tier 2). If speakers in a conversational context – as opposed to read speech – increase the strength of language-general (acoustic-phonetic) cues to compensate for weak language-specific (semantic and phonotactic) cues, this may result in divergent patterns of segmentation behavior for the two styles. However, if perceptual results with conversational speech mirror those with read speech, we could then conclude either (a) that any speaker-driven cue trade-offs in conversational speech are not of sufficient salience to affect listeners’ segmentation behavior, or (b) that such trade-offs are equally present in read and conversational speech. Either of these conclusions would support the ecological validity of the extensive body of speech segmentation literature based on read speech.

Using standard laboratory speech methodology, Mattys et al. (2005) identified three tiers in a hierarchical segmentation framework. The lexical level (Tier 1) is based on listeners’ knowledge of individual words and of syntactic, semantic, and pragmatic relations between words. The sub-lexical level is divided into acoustic-segmental cues (Tier 2: e.g., phonotactics, coarticulation, allophony, initial, and final lengthening) and metrical prosody (Tier 3: specifically the placement of lexical stress).

Within that framework, reliance on Tier 1 information is contingent on language-specific experience. For example, the presence of a known word within the speech string allows listeners to infer boundaries immediately preceding or following it, sometimes referred to as “segmentation-by-lexical-subtraction” (e.g., Dahan and Brent, 1999; Mattys et al., 2005; White et al., 2010a). Syntactic structure and semantic context also guide listeners to the appropriate segmentation solution (e.g., Blank and Foss, 1978; Tyler and Wessels, 1983; Mattys et al., 2007).

Listeners similarly require language-specific experience to utilize certain cues at the sub-lexical level. For example, a pre-requisite for the exploitation of phonotactic segmentation cues is knowledge of language-specific statistics on the occurrence of segmental sequences within and across syllable and word boundaries (e.g., McQueen, 1998; Mattys et al., 2005). Likewise, in Tier 3, the use of metrical stress for segmentation requires familiarity with a language’s predominant stress pattern (e.g., Cutler and Carter, 1987; Vroomen and de Gelder, 1995). However, other cues, particularly within Tier 2, appear to arise from language-universal mechanisms through which word and phrase boundaries are made acoustically salient by speakers (e.g., Tyler and Cutler, 2009). In particular, boundary-adjacent gestural strengthening and segmental lengthening have been observed in many of the world’s languages (e.g., Keating et al., 2003).

Mattys et al. (2005) found that listeners’ use of cues depends on interpretive conditions, with Tier 1 cues exploited, when available, in preference to sub-lexical sources of information. Tier 2 cues are used when lexicality and linguistic context fail to provide an unambiguous guide to segmentation. In English, metrical stress (Tier 3) has the lowest weight, and is only relied upon when acoustic-segmental cues are made inaccessible or unreliable, for example, in noisy listening conditions.

As discussed above, the H&H hypothesis leads naturally to the prediction that where language-specific cues (lexicality, semantics, phonotactics, etc.) strongly indicate a segmentation solution, language-general acoustic-phonetic cues (those contingent on the speaker’s articulatory effort) are likely to be minimized. This modulation of acoustic-phonetic cues is also congruent with information-driven accounts of speech timing, such as the smooth signal redundancy hypothesis (e.g., Aylett and Turk, 2004, 2006). In this approach, the rate of information flow within the signal is kept approximately constant by expanding phonetic material at points of low redundancy. As information regarding linguistic structure, such as the placement of word boundaries, is central to the calculation of the redundancy profile, segmental lengthening at unpredictable boundaries is a natural corollary (see also Turk, 2010).

In this study, we compared the exploitation of segmentation cues in materials from conversational speech and from read speech. Given that conversational speech tends to be generally less intelligible (e.g., Pickett and Pollack, 1963; Duez, 1995), we expected to find that segmentation is overall a more difficult task with conversational speech than read speech. We also tested the hypothesis that, in conversational speech, the perceptual strength of language-general acoustic-phonetic cues is modulated by the availability of language-specific cues, focusing on semantic predictability (Tier 1 in the Mattys et al., 2005, segmentation hierarchy), and phonotactic frequency (Tier 2).

With regard to semantics, the H&H hypothesis leads to the prediction that speakers should minimize articulatory effort to indicate word-boundary cues when semantic context strongly favors a particular segmentation solution. Thus, acoustic-phonetic cues should be weaker in a semantically plausible phrase such as *oil tanker* compared with an unlikely phrase like *seal tanker*. Such a trade-off is more likely in spontaneous speech, where the speaker has a clear communicative goal, than in read speech. Indeed, the H&H approach presupposes that the ultimate goal of the speaker’s articulation is comprehension by the listener (e.g., Lindblom, 1996) – thus, modulation of acoustic-phonetic cues should be evident in listeners’ segmentation behavior, potentially even when Tier 1 cues are available.

A similar trade-off would be expected within Tier 2 of the hierarchy. For example, where a collocation of words provides little phonotactic evidence of an intervening boundary, e.g., *drab rickshaw* (the cross-boundary /br/ being a high within-word frequency diphone), the speaker should maximize acoustic-phonetic segmentation cues for listeners; in contrast, acoustic-phonetic cues would be minimal in the presence of strong phonotactic cues, e.g., *cream rickshaw* (/mr/ being a low within-word frequency diphone). Once again, such a trade-off may result in differential cue reliance by listeners to spontaneous and read speech.

## Materials and Methods

### Participants

The participants were 224 native speakers of British English, self-reported as having no hearing or speech difficulties. They were undergraduates of the University of Bristol and received course credit or a small honorarium for their participation.

### Materials

To elicit our stimuli, we devised a map task in which pairs of speakers interacted conversationally regarding routes around stylized landmarks (White et al., 2010b; White et al., in preparation). Landmark names were one- or two-word phrases in which the strength of potential segmentation cues was systematically varied. Of relevance to the current experiment are the semantics and phonotactics conditions.

#### Semantics condition

The frequency with which the two words of the landmark co-occur in natural spoken language was systematically varied within landmark pairs: for example, *oil tanker* has a high word bigram frequency and *seal tanker* has a low word bigram frequency. The frequencies of occurrence of each word bigram were derived from the British National Corpus, a 100 million word collection of samples of written and spoken language (British National Corpus, 2007). The full set of phrases used in the current experiment, including BNC counts and *z*-scores, are shown in Table 1. The *z*-score adjusts for the relative frequencies of the individual words of the bigram, and so indicates how likely the second word is given the first (British National Corpus, 2007).

**Table 1:** **Phrase pairs used in the semantics condition**.

Low predictability			High predictability		
	BNC count	*z*-score		BNC count	*z*-score
*Seal tanker*	0	–	*Oil tanker*	36	186
*Burning alley*	0	–	*Bowling alley*	24	397
*Breakfast march*	0	–	*Protest march*	41	55
*Garlic bag*	0	–	*Plastic bag*	207	465
*German mill*	0	–	*Cotton mill*	36	134
*Burning chair*	0	–	*Rocking chair*	51	298
*Runner jacket*	0	–	*Dinner jacket*	42	109

*The number of occurrences and the *z*-score for each phrase are derived from the British National Corpus (∼100M words)*.

Landmark phrases had three possible syllable structures: monosyllable-disyllable (1–2, e.g., *seal tanker*); disyllable-monosyllable (2–1, e.g., *garlic bag*); disyllable-disyllable (2–2, e.g., *dinner jacket*). Syllable structures were matched within phrase pairs.

#### Phonotactics condition

In the phonotactics condition, pairs of landmarks were devised to contrast in the frequency with which their cross-boundary diphone occurs within and between words: for example, /mr/ in *cream rickshaw* is more common between words than within words, whilst /br/ in *drab rickshaw* is more common within words than between words (see Table 2). In this condition, all experimental landmark phrases had a 1–2 syllable structure.

**Table 2:** **Within-word and between-word diphone statistics calculated from the Buckeye corpus for the segmentation-favorable and segmentation-unfavorable diphone pairs in the phonotactics condition**.

	Diphone	Buckeye counts				Between/within ratio	Favorable/unfavorable index
		Within-word		Between-word	
		Raw	Norm	Raw	Norm	
*Cream rickshaw*	/mr/	0	0.00	108	0.57	323	12285
*Drab rickshaw*	/br/	455	0.81	4	0.02	0.03	
*Mauve tiger*	/vt/	0	0.00	623	3.31	1862	11789
*Swiss tiger*	/st/	8137	14.48	430	2.29	0.16	
*Swiss ruler*	/sr/	20	0.04	250	1.33	37	2492
*Half ruler*	/fr/	595	2.84	8	0.04	0.02	
*Cream candle*	/mk/	0	0.00	143	0.76	427	2193
*Long candle*	/Nk/	2132	3.79	139	0.74	0.20	
*Cream lipstick*	/ml/	27	0.05	200	1.06	22	1781
*Drab lipstick*	/bl/	962	1.71	4	0.02	0.01	
*Hot cannon*	/tk/	2	0.00	822	4.37	1229	1618
*Swiss cannon*	/sk/	1248	2.22	317	1.69	0.76	
*Hot chalet*	/tS/	0	0.00	289	1.54	864	1243
*Black chalet*	/kS/	215	0.38	50	0.27	0.70	

*The first landmark in each pair contains the segmentation-favorable diphone. Diphones are given in SAMPA transcription*.

The within-word and between-word frequencies were calculated from the Buckeye corpus of American English conversational speech (Pitt et al., 2007). We converted the orthographic transcription of the entire Buckeye corpus to phonemic transcriptions using the Celex database dictionary (Baayen et al., 1995). From our phonemic transcription of the Buckeye corpus, we derived a list of all diphone sequences (i.e., any set of two phonemes that occurred in sequence at least once) and calculated the number of occurrences of each diphone within words and between words (i.e., spanning a word boundary).

There were 1075 diphone types that occurred within words, and 604434 total occurrences of these diphones. Thus the average within-word token/type ratio was 562. For between-word diphones, there were 1190 types and a total of 224040 occurrences, making an average between-word token/type ratio of 188. These ratios were used to normalize the counts for the individual within-word and between-word diphones (e.g., for /br/, the within-word count is 455, and so the normalized count is 455/562 = 0.81). Raw and normalized counts are shown in Table 2 for the selected diphone pairs. (For some diphones, there were no Buckeye examples in within-word context, so we used a count of 1 to derive the normalized count, 0.0018.) We further calculated ratios of normalized counts between/within words, as an indication of how favorable each diphone is for word segmentation.

Finally, a favorable/unfavorable diphone index, shown in the final column of Table 2, was derived by dividing the within/between-word ratio for the segmentation-favorable diphones by the within/between-word ratio for the segmentation-unfavorable diphones. Thus, for example, the diphone /mr/ was never heard within words in the Buckeye corpus, but there were 108 instances between words. In contrast, the diphone /br/ was heard 455 times within words and only four times between words. From these statistics, the favorable/unfavorable index of 12285 indicates that /mr/ is an overwhelmingly stronger cue to an intervening word boundary.

#### Recordings of spontaneous speech and read speech

**Figure 1:**
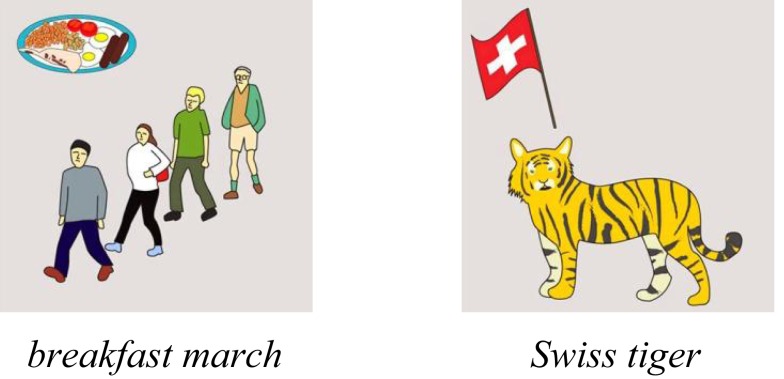
**Examples of the landmark pictograms used in the spontaneous speech map task**.

Test landmarks were represented by two-element pictograms (e.g., Figure 1). In a preliminary familiarization session, participants were trained to recognize and name: (i) the individual landmark elements, (ii) the composite pictograms. This training, which required at most three exposures in each phase, was carried out a few days before the map task dialogs were recorded.

The full set of landmarks was distributed over eight base maps, which each contained 20 landmarks in total. Phrases within a contrasting pair (e.g., *cream rickshaw* vs. *drab rickshaw* or *oil tanker* vs. *seal tanker*) were never used in the same map.

Each pair of interlocutors was presented with two sets of eight maps (i.e., two different versions of the same eight base maps, distinguished by having the landmarks in different spatial arrangements and distinct routes marked around the landmarks). For each map dialog, one interlocutor – the “describer” – had a full map including a marked route, whilst the other – the “follower” – had the same map without the marked route. The describer and the follower could see each other’s faces, but not the other map, and the task was for the describer to guide the follower around the route. After eight maps, the describer and the follower switched roles and then interacted on another eight maps.

Utterances from these map dialogs in which at least one landmark was mentioned were transcribed. Each speaker then returned on a later occasion – at least 1 month after the initial recording – to re-record the selected utterances. This time they simply read a list of sentences (the utterance transcriptions) in random order without any dialog context. Thus, we generated two parallel sets of stimuli in contrasting speech styles (“spontaneous” vs. “read”).

Eight pairs of speakers were recorded for the spontaneous map task and the read sentences.

#### Extraction of the experimental stimuli

From the full set of recordings for the spontaneous and read corpora, we extracted four sets of landmark repetitions for the semantics condition and four sets for the phonotactics condition. These sets were based on the landmark utterances produced by seven of the 16 speakers, selected from those speakers who produced all relevant landmarks without error on the first mention. Each set comprised 28 landmarks: i.e., spontaneous versions and read versions of the seven pairs of landmarks (Table 1 – semantics condition; Table 2 – phonotactics condition).

Within a set, the same speaker produced the spontaneous and read versions of each landmark pair, with the seven landmark pairs read by different speakers (e.g., one speaker produced the two versions – spontaneous/read – of *oil tanker* and *seal tanker*, whilst another speaker produced the two versions of *plastic bag* and *garlic bag*, etc.). Thus, all seven speakers and all seven landmarks pairs were represented in a set. Landmark-speaker pairings were varied between the four sets in each condition. All landmark tokens represented the first utterance by that speaker of that landmark name.

The mean loudness of landmark phrases was normalized within the two conditions (phonotactics and semantics). The durations of the onset consonants and stressed vowels of the second word of landmark phrases (Tables 1 and 2) were measured by visual inspection in Praat (Boersma and Weenink, 2006) according to standard criteria (Turk et al., 2006), as was the total duration of the second word. Both word-initial lengthening, indexed by onset consonant duration, and lexical stress, indexed by stressed vowel duration, are potential cues to segmentation (e.g., Mattys et al., 2005, 2007), whilst variation in total word duration provides a guide to the degree of phrasal stress on the second word of the landmarks (words without phrasal stress may be less salient and thus less effective primes). Shorter word durations could also be indicative of reduction processes in spontaneous speech, which have been shown to reduce the efficacy of semantic primes with a short interstimulus interval (ISI; van de Ven et al., 2011). Durational analyses are reported below for the two conditions.

### Design and procedure

We used cross-modal identity priming to visual lexical decision to assess listeners’ segmentation behavior, a similar procedure to that used in earlier segmentation studies (e.g., Mattys et al., 2005). On each trial, participants heard a landmark phrase played over headphones, and then saw a letter string – the target – presented in the center of a computer screen. Participants were required to make a lexical decision to the letter string, pressing the right shift key if they thought it was an English word and the left shift key otherwise.

The letter string appeared immediately after the offset of the landmark phrase. A delay of 100 ms has been determined in previous studies to be most effective in allowing priming effects to be observed in lexical decision (Mattys, 2004). However, this interval was established with two-syllable fragment primes for three-syllable targets, whereas in the present experiment, we used two-syllable full word primes. Thus, because of the full overlap between prime and target, we eliminated the ISI in order to maintain focus on online segmentation, a principled compromise given that Mattys, 2004) also showed strong priming effects with a 0 ms ISI. There was a 3-s interval following a participant’s response before the onset of the next trial, with a response time-out of 10 s.

The design for both the semantics and the phonotactics conditions is summarized in Table 3. There were 136 trials for each participant. In the 28 experimental trials, the visual target was the second word of the landmark prime: e.g., landmark phrase *cream rickshaw* → visually presented “RICKSHAW.”

**Table 3:** **Overview of the 136 cross-modal identity priming trials**.

Priming: *landmark phrase* *→* “TARGET”	“TARGET” lexical status	
	Word	Non-word
Related	28 Experimental trials, e.g.,: *cream rickshaw* *→* “RICKSHAW”	44 Fillers, e.g.,: *snow valley* *→* “SNOEVAL”
		*winner jacket* *→* “NERJACK”
	16 Fillers, e.g.,: *flounder boat* *→* “FLOUNDER”	*timber plate* *→* “BUPLEYT”
Unrelated	24 Fillers, e.g.,: *chain anchor* *→* “FIGTREE”	24 Fillers, e.g.,: *dry tanker* *→* “LOESHAL”

*Specific examples are from the semantics condition. For related trials, examples illustrate the variable overlap between landmark phrase and target*.

The remaining trials were fillers, selected so that the total of 68 experimental and filler trials with word targets were balanced by 68 filler trials with non-word targets. As shown in Table 3, there were 88 trials in which landmark phrase and target were related and 48 trials in which they were unrelated. In related filler trials, the overlap between landmark phrases and targets was varied, so that the target could be from the initial, medial, or final part of the landmark (in experimental trials, the target always related to the final one or two syllables of the landmark). So that specific landmarks were not reliable predictors of lexicality, there were 10 landmark phrases in the related condition that were used once with a word target and once with a non-word target. In the unrelated conditions, there were 23 landmark phrases that were repeated between word and non-word targets.

In the semantics condition, as described above, four of the seven experimental target types were monosyllabic and the other three were disyllabic; filler targets (word and non-word) could also be either monosyllabic or disyllabic. In the phonotactics condition, all experimental and fillers targets were disyllabic.

In the experimental trials within each condition, equal numbers of participants heard each landmark set (see previous section): 26 participants per set for semantics, 30 per set for phonotactics. For the filler landmarks, the seven speakers were represented approximately equally, with tokens taken from both spontaneous and read recordings.

Participants heard each experimental landmark twice, once as spontaneous speech and once as read speech. The majority of filler landmarks were also heard twice, but to increase variability, there were a few filler landmarks with three or four repetitions. The seven experimental targets were repeated four times (spontaneous/read × favorable/unfavorable), balanced by seven non-word filler targets also repeated four times. Other word and non-word targets were seen once or twice.

The trials were distributed over four randomized blocks, with the order of blocks varied between participants. Participants never heard the same landmark phrase or saw the same experimental target more than once in the same block.

## Results

Lexical-decision latencies were measured from the onset of visual target presentation. We excluded participants whose mean latencies in the experimental trials were more than two standard deviations greater than the overall participant mean within their own condition (semantics or phonotactics). There were three such participants excluded for each condition, leaving 101 participants in the semantics condition and 117 in the phonotactics condition.

Table 4 shows the lexical-decision accuracy (%) by condition. Analyses of accuracy data are reported below for each condition.

**Table 4:** **Proportion of correct lexical decisions (%) by condition**.

	Spontaneous		Read	
	Favorable	Unfavorable	Favorable	Unfavorable
Semantics	99	99	98	97

For the analyses of lexical-decision latencies, incorrect responses to word targets and correct responses 2 standard deviations from the mean latency were discarded on a participant-by-participant basis. The proportion of data thus discarded was 7% for the semantics condition and 11% for the phonotactics condition.

**Figure 2:**
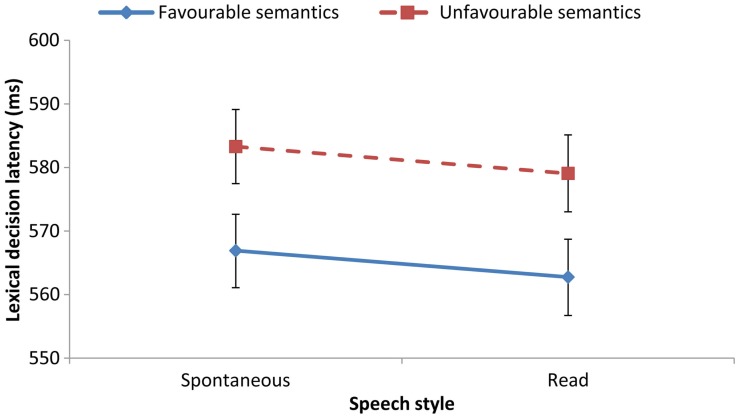
**Lexical-decision latencies (and standard errors) in the semantic segmentation condition for the two speech styles: spontaneous map dialogs vs. read sentences (101 participants)**. Favorable semantic cues: High word bigram frequency (e.g., oil tanker). Unfavorable semantic cues: Low word bigram frequency (e.g., *seal tanker*).

**Figure 3:**
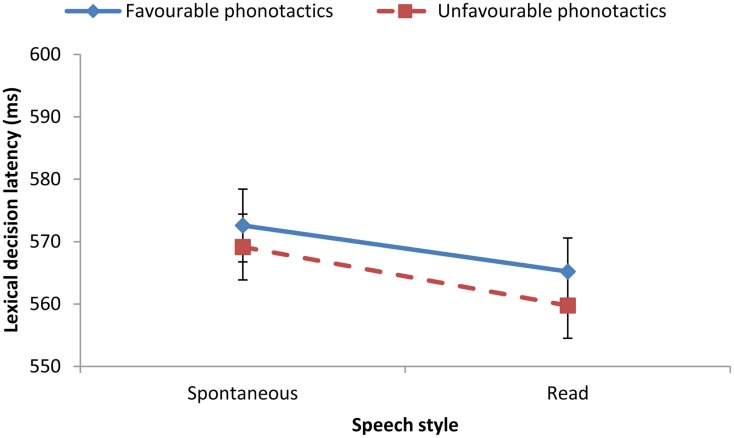
**Lexical-decision latencies (and standard errors) in the phonotactic segmentation condition for the two speech styles: spontaneous map dialogs vs. read sentences (117 participants)**. Favorable phonotactic cues: low within-word frequency of cross-boundary diphone (e.g., *cream rickshaw*). Unfavorable phonotactic cues: high within-word frequency of cross-boundary diphone (e.g., *drab rickshaw*).

We then constructed a series of mixed-effect regression models with predictive factors of Cue (semantics vs. phonotactics), Cue Valence (favorable vs. unfavorable for segmentation), and Speech Style (spontaneous map dialogs vs. read sentences), and with random factors of participants and test items (random intercepts only). Mean latencies are shown in Figure 2 for the Semantics condition and Figure 3 for the Phonotactics condition.

Comparing mixed-effect regression models with and without these predictive factors indicated that there was a main effect of Style: χ^2^ (1) = 5.52, *p* < 0.05; thus lexical-decision latencies were longer when primes were extracted from spontaneous speech than from read speech. There was no main effect of Cue: χ^2^ (1) = 0.06, *p* > 0.10; thus mean latencies were similar for the semantics and phonotactics condition.

There was a near-significant effect of Valence: χ^2^ (1) = 2.94, *p* = 0.086, and a significant interaction between Cue and Valence: χ^2^ (1) = 10.03, *p* < 0.005, both explored further below. The interactions Valence × Style and Cue × Style, and the three-way interaction Valence × Cue × Style did not approach significance. Given the Cue × Valence interaction, as well as previous findings regarding differential exploitation of semantics and phonotactics, we further examined segmentation behavior in separate analyzes for the two cues.

### Results: Semantics

As indicated by a main effect of Valence, χ^2^ (1) = 8.95, *p* < 0.005, lexical-decision latencies were faster when the preceding spoken utterance contained semantically favorable segmentation cues, i.e., high-frequency word pairs such as *oil tanker*, than unfavorable cues, i.e., low-frequency word pairs such as *seal tanker* (Figure 2). This effect is in line with previous findings that the predictability of high-frequency word sequences facilitates segmentation and hence priming of the visual target (Mattys et al., 2005). There was no effect of Style, χ^2^ (1) = 2.43, *p* > 0.10 and no interaction between Valence and Style, χ^2^ (1) = 0.05, *p* > 0.10.

Thus, despite the expectation that landmarks in the spontaneous dialogs may have been hypoarticulated compared with the read utterances, priming did not differ. To explore the acoustic-phonetic modulation hypothesis, we analyzed the durational data for the semantics condition (Table 5). For onset consonant duration, there was a main effect of Style, χ^2^ (1) = 7.15, *p* < 0.01, with onsets longer in the read condition than the spontaneous condition. There was also a main effect of Valence, χ^2^ (1) = 4.92, *p* < 0.05, with longer onsets in the unfavorable landmarks. There was no interaction between Style and Valence, χ^2^ (1) = 0.04, *p* > 0.10. For both stressed vowel duration and whole word duration, there was no effect of Style, no effect of Valence and no interaction between Style and Valence, all *p*s > 0.10.

**Table 5:** **Mean durations in milliseconds (standard errors in brackets) for constituents of the second word of landmark phrases in the semantics condition**.

	Spontaneous		Read	
	Favorable	Unfavorable	Favorable	Unfavorable
Onset consonant	78 (5)	85 (6)	87 (6)	94 (6)
Stressed vowel	143 (12)	143 (10)	141 (14)	150 (14)
Whole word	389 (43)	392 (38)	386 (39)	400 (32)

*E.g., *tanker* in *oil tanker* (favorable) vs. *seal tanker* (unfavorable)*.

The analyses of onset consonant duration do indeed suggest hypoarticulation of segmentation cues in spontaneous speech compared with read speech (Table 5). There is also evidence of cue trading in both spontaneous and read speech, with longer onset consonants in landmark phrases where semantics does not serve to promote segmentation (e.g., *seal tanker* compared with *oil tanker*). However, the priming data provided no evidence that listeners exploited these subtle modulations for segmentation, but accorded instead with the semantic predictability of the landmark.

To determine if the priming pattern could have resulted from a speed-accuracy trade-off, we analyzed the accuracy data (Table 4). There was an effect of Style, χ^2^ (1) = 6.67, *p* < 0.01, indicating more errors with read than spontaneous stimuli. There was no effect of Valence, χ^2^ (1) = 1.49, *p* > 0.10, and no interaction between Style and Valence, χ^2^ (1) = 1.50, *p* > 0.10. Thus, although there was a small difference between speaking styles in terms of accuracy, there was no evidence that greater priming in the favorable condition was the consequence of lower accuracy.

### Results: Phonotactics

In contrast with semantics, there was no effect of Valence in the phonotactics condition, χ^2^ (1) = 0.72, *p* > 0.10, but there was a main effect of Style, χ^2^ (1) = 4.18, *p* < 0.05 (Figure 3). There was no interaction between Valence and Style, χ^2^ (1) = 0.61, *p* > 0.10.

The lack of Valence effect suggests that the cross-boundary phonotactic characteristics of the landmark phrases did not affect lexical-decision latency to the visual target. Thus, despite the high statistical power afforded by 117 participants, we found no evidence of the impact of phonotactics on segmentation.

Although relatively small, the effect of Style offers a further contrast with the semantics condition: Lexical-decision latencies were faster when the visual target followed a phrase extracted from read sentences rather than from spontaneous dialogs. This result accords with the general hypothesis for spontaneous speech, i.e., subtle acoustic-phonetic cues to word boundaries may be more salient in read speech than in conversational speech.

We assessed the durational evidence for modulation by speakers of acoustic-phonetic cues in the phonotactics condition (Table 6). For the duration of the onset consonant, there was no effect of Style, no effect of Valence, and no interaction between Valence and Style, all *p*s > 0.10. For the stressed vowel, there was no effect of Style, χ^2^ (1) = 0.01, *p* > 0.10, but there was a near-significant effect of Valence, χ^2^ (1) = 2.88, *p* = 0.09, and a near-significant interaction between Style and Valence, χ^2^ (1) = 2.89, *p* = 0.09. For the total duration of the second word of the landmark, there was no effect of Style, no effect of Valence, and no interaction, all *p*s > 0.10.

**Table 6:** **Mean durations in milliseconds (standard errors in brackets) for constituents of the second word of landmark phrases in the phonotactics condition: e.g., *rickshaw* in *cream rickshaw* (favorable) vs. *drab rickshaw* (unfavorable)**.

	Spontaneous		Read	
	Favorable	Unfavorable	Favorable	Unfavorable
Onset consonant	88 (5)	92 (5)	90 (5)	97 (6)
Stressed vowel	91 (7)	103 (8)	98 (7)	98 (7)
Whole word	433 (22)	449 (21)	428 (19)	437 (19)

Thus, the durational data cannot account for the effect of Style on lexical-decision latencies. There was some evidence of cue trading by speakers in the spontaneous condition, with longer post-boundary stressed vowels with unfavorable landmarks (e.g., the primary stressed vowel in *rickshaw* was longer in *drab rickshaw* than *cream rickshaw* – see Table 6). Such cue trading in spontaneous speech is in keeping with the H&H hypothesis. However, this acoustic-phonetic variation did not affect listeners’ segmentation behavior. Thus, from a perceptual viewpoint, these data reinforce our conclusion from the semantics condition: Contrary to the prediction based on the H&H hypothesis, speaker-driven segmentation cue trade-offs in spontaneous speech were not exploited by listeners, at least when lexical segmentation cues were available.

Accuracy analyses (Table 4) did not indicate any effect of Valence or Style, nor any interaction, all *p*s > 0.10.

## Discussion

We used cross-modal identity priming to examine listeners’ use of semantic and phonotactic segmentation cues in conversational vs. read speech. We found clear support for the use of semantics, but not for the use of phonotactics. There was some evidence from the phonotactics condition that segmentation was more difficult overall with conversational speech than with read speech, but this finding did not generalize to the semantics condition. There was no evidence that listeners to conversational or read speech paid attention to speaker-driven modulation – based on semantic or phonotactic cue strength – of acoustic-phonetic cues.

The semantics result accords with Mattys et al.’s (2005) hierarchical segmentation framework insofar as it confirm the primacy of Tier 1 cues to word boundaries. It should be noted that the exact mechanism responsible for the semantic effect cannot be fully ascertained in this experiment: It may be that listeners benefited from semantic priming (e.g., auditory *oil* primes visual *tanker*) or identity priming (e.g., auditory *tanker* primes visual *tanker*), or a combination of both. In any case, however, faster lexical decision indicates an influence of foregoing landmark phrases and hence easier segmentation of individual words from the phrase. All experimental stimuli were, however, taken from the first mentions by speakers of the particular landmarks: In more hypoarticulated speech, such as is possible with subsequent repetitions of landmarks, there may be some attenuation of semantic priming (van de Ven et al., 2011).

The failure to find a phonotactic segmentation effect, despite the large number of participants, presents an apparent contrast with some previous findings, but in fact phonotactic effects have been demonstrated only when a complete lexical segmentation solution for the phrase was unavailable (McQueen, 1998; Mattys et al., 2005). For example, in McQueen and in Mattys et al. (Experiment 2), the target word/part-word was preceded or followed by a nonsense string. Similarly, in Mattys et al. Experiments 4 and 5, the phonotactic effect only emerged when lexical or semantic cues were removed by truncating the prior context. Thus, we conclude – in line with the hierarchical framework – that listeners’ use of phonotactics for segmentation is fragile and only manifest when lexical cues are lacking. Likewise, Newman et al. (2011) found that probabilistic phonotactic constraints – in that case, the rareness of syllable-final lax vowels in English – did not affect segmentation.

There was some evidence, both from perception and production, that acoustic-phonetic cues may be attenuated slightly in conversational speech. First, in the semantic condition, word-initial onset lengthening appeared stronger in read than spontaneous speech. Second, in the phonotactics condition, lexical-decision latencies were longer with spontaneous speech tokens. The lack of congruence between the durational and perceptual data indicates that listeners in the phonotactics condition responded to acoustic-phonetic differences between speech styles not captured by the timing analyses. We note that the read speech priming advantage in the phonotactics condition does concur with findings that listeners are highly sensitive to acoustic-phonetic cues in tasks where cues such as phonotactics are ignored (Fernandes et al., 2010; Newman et al., 2011).

Of course, the small reduction in priming from the conversation-derived tokens in the phonotactics condition may reflect the overall reduced intelligibility of conversational speech (e.g., Pickett and Pollack, 1963) rather than segmentation *per se*. However, the landmark words priming the targets in the phonotactics condition were all disyllables, which should suffer less of an overall intelligibility drop due to hyperarticulation than the mixture of monosyllables and disyllables in the semantics condition. Furthermore, differences in second landmark word duration were minimal, not statistically robust, and did not consistently reflect the priming patterns. More work would be required to resolve the locus of the difference between read and conversational styles, which is in any case small and inconsistent between conditions.

Importantly, the absence of interactions between cue strength and speech style in segmentation behavior is contrary to strong versions of theories in which the conversational speaker’s articulation is directly commensurate with listeners’ needs, e.g., H&H (Lindblom, 1990, 1996). However, the durational analyses suggest that speakers may actively trade-off acoustic-phonetic cues and cues from Tier 1 (semantics) or Tier 2 (phonotactics), particularly in spontaneous speech, but that this modulation of acoustic-phonetic cues is below a level that is exploitable by listeners when presented with phrases for which lexicality provides a segmentation solution. H&H-type cue trading may become perceptually important in more difficult listening conditions.

With reference to the hierarchical segmentation framework of Mattys et al. (2005), we conclude that listeners’ use of language-specific cues to word boundaries is consistent in the case of Tier 1 cues, such as semantics, and rather evanescent in the particular case of phonotactics, a Tier 2 cue. Furthermore, there is no evidence from the present data that, in natural conversation, speakers modulate their word-boundary articulation according to the availability of language-specific segmentation cues to a degree that directly influences speakers’ segmentation behavior when confronted with clear, lexically intact phrases. The hierarchical framework suggests that such speaker-driven cue trading may be more effective for listeners where lexical segmentation is problematic. Indeed, a next step would be to consider whether any cue trade-offs that speakers produce play a role for listeners when confronted with the ambiguity, variability, and environmental intrusion that natural dialog contexts may entail.

## Conflict of Interest Statement

The authors declare that the research was conducted in the absence of any commercial or financial relationships that could be construed as a potential conflict of interest.

## References

[B1] Aylett M. Turk A. (2004). The smooth signal redundancy hypothesis: a functional explanation for relationships between redundancy, prosodic prominence, and duration in spontaneous speech. Lang. Speech 47, 31–56 10.1177/00238309040470010201 15298329

[B2] Aylett M. Turk A. (2006). Language redundancy predicts syllabic duration and the spectral characteristics of vocalic syllable nuclei. J. Acoust. Soc. Am. 119, 3048–3058 10.1121/1.2188331 16708960

[B3] Baayen H. R. Piepenbrock R. Gulikers L. (1995). The CELEX Lexical Database. Release 2 (CD-ROM). Philadelphia: Linguistic Data Consortium, University of Pennsylvania

[B4] Blank M. A. Foss D. J. (1978). Semantic facilitation and lexical access during sentence processing. Mem. Cognit. 6, 644–652 10.3758/BF03198255

[B5] Boersma P. Weenink D. (2006). Praat: Doing Phonetics by Computer (Version 4.4.24) [Computer Program]. Available at: http://www.praat.org/ [accessed June 19, 2006]

[B6] British National Corpus, version 3 (BNC XML Edition) (2007). Distributed by Oxford University Computing Services on behalf of the BNC Consortium. Available at: http://www.natcorp.ox.ac.uk/

[B7] Brown G. (1977). Listening to Spoken English. Harlow: Longman

[B8] Cutler A. Carter D. M. (1987). The predominance of strong initial syllables in the English vocabulary. Comput. Speech Lang. 2, 133–142 10.1016/0885-2308(87)90004-0

[B9] Dahan D. Brent M. R. (1999). On the discovery of novel wordlike units from utterances: an artificial-language study with implications for native-language acquisition. J. Exp. Psychol. Gen. 128, 165–185 10.1037/0096-3445.128.2.165 10406104

[B10] Duez D. (1995). On spontaneous French speech: aspects of the reduction and contextual assimilation of voiced stops. J. Phon. 23, 407–427 10.1006/jpho.1995.0031

[B11] Fernandes T. Kolinsky R. Ventura P. (2010). The impact of attention load on the use of statistical information and coarticulation as speech segmentation cues. Atten. Percept. Psychophys. 72, 1522–1532 10.3758/APP.72.6.1522 20675798

[B12] Keating P. A. Cho T. Fougeron C. Hsu C. (2003). “Domain-initial strengthening in four languages,” in Phonetic Interpretation: Papers in Laboratory Phonology Vol. 6, eds Local J. Ogden R. Temple R. (Cambridge: Cambridge University Press), 145–163

[B13] Klatt D. H. Stevens K. N. (1973). On the automatic recognition of continuous speech. Implications of a spectrogram-reading experiment. IEEE Trans. Acoust. 21, 210–217

[B14] Lindblom B. (1990). “Explaining phonetic variation: a sketch of the H&H theory,” in Speech Production and Speech Modelling, ed. Hardcastle W. J. Marchal A. (Amsterdam: Kluwer), 403–439

[B15] Lindblom B. (1996). Role of articulation in speech perception: clues from production. J. Acoust. Soc. Am. 99, 1683–1692 10.1121/1.414691 8819859

[B16] Mattys S. L. (2004). Stress versus coarticulation: toward an integrated approach to explicit speech segmentation. J. Exp. Psychol. Hum. Percept. Perform. 30, 397–408 10.1037/0096-1523.30.2.397 15053697

[B17] Mattys S. L. Melhorn J. F. White L. (2007). Effects of syntactic expectations on speech segmentation. J. Exp. Psychol. Hum. Percept. Perform. 33, 960–977 10.1037/0096-1523.33.4.960 17683240

[B18] Mattys S. L. White L. Melhorn J. F. (2005). Integration of multiple speech segmentation cues: a hierarchical framework. J. Exp. Psychol. Gen. 134, 477–500 10.1037/0096-3445.134.4.477 16316287

[B19] McQueen J. M. (1998). Segmentation of continuous speech using phonotactics. J. Mem. Lang. 39, 21–46 10.1006/jmla.1998.2568

[B20] Mehta G. Cutler A. (1988). Detection of target phonemes in spontaneous and read speech. Lang. Speech 31, 135–156 3256770 10.1177/002383098803100203

[B21] Newman R. S. Sawusch J. R. Wunnenberg T. (2011). Cues and cue interactions in segmenting words in fluent speech. J. Mem. Lang. 64, 460–476 10.1016/j.jml.2010.11.004

[B22] Pickett J. M. Pollack I. (1963). Intelligibility of excerpts from fluent speech: effects of rate of utterance and duration of excerpt. Lang. Speech 6, 151–164

[B23] Pitt M. A. Dilley L. Johnson K. Kiesling S. Raymond W. Hume E. (2007). Buckeye Corpus of Conversational Speech (2nd Release). Columbus, OH: Department of Psychology, Ohio State University [Distributor].

[B24] Turk A. (2010). Does prosodic constituency signal relative predictability? A smooth signal redundancy hypothesis. Lab. Phonol. 1, 227–262

[B25] Turk A. Nakai S. Sugahara M. (2006). “Acoustic segment durations in prosodic research: a practical guide,” in Methods in Empirical Prosody Research, eds Sudhoff S. Lenertova D. Meyer R. Pappert S. Augurzky P. Mleinek I. Richter N. Schliesser J. (Berlin: de Gruyter), 1–28

[B26] Tyler L. K. Wessels J. (1983). Quantifying contextual contributions to word-recognition processes. Percept. Psychophys. 34, 409–420 10.3758/BF03203056 6657445

[B27] Tyler M. D. Cutler A. (2009). Cross-language differences in cue use for speech segmentation. J. Acoust. Soc. Am. 126, 367–376 10.1121/1.3129127 19603893 PMC2723901

[B28] van de Ven M. Tucker B. Ernestus M. (2011). Semantic context effects in the comprehension of reduced pronunciation variants. Mem. Cognit. 39, 1301–1316 10.3758/s13421-011-0103-2 21547604

[B29] Vroomen J. de Gelder B. (1995). Metrical segmentation and lexical inhibition in spoken word recognition. J. Exp. Psychol. Hum. Percept. Perform. 21, 98–108 10.1037/0096-1523.21.1.98

[B30] White L. Melhorn J. F. Mattys S. L. (2010a). Segmentation by lexical subtraction in Hungarian L2 speakers of English. Q. J. Exp. Psychol. (Hove) 63, 544–554 10.1080/17470210903006971 19591079

[B31] White L. Wiget L. Rauch O. Mattys S. L. (2010b). “Segmentation cues in spontaneous and read speech,” in Proceedings of the Fifth Conference on Speech Prosody 2010, Chicago, 100218: 1–4

